# Seeking connection: A qualitative study of psychosocial support needs of rural cancer survivors in Minnesota

**DOI:** 10.1111/jrh.70066

**Published:** 2025-08-04

**Authors:** Morgan Gruner, Katherine Brown, Renee Anderson, Shaunequa James, Xuan Li, Carrie Henning‐Smith, Anne Blaes, Patricia Jewett, Rachel I. Vogel

**Affiliations:** ^1^ Department of Obstetrics, Gynecology, and Women's Health University of Minnesota Minneapolis Minnesota USA; ^2^ Gilda's Club Minnetonka Minnesota USA; ^3^ Division of Health Policy and Management University of Minnesota Minneapolis Minnesota USA; ^4^ Division of Hematology, Oncology and Transplantation University of Minnesota Minneapolis Minnesota USA; ^5^ Division of Environmental Health Sciences University of Minnesota Minneapolis Minnesota USA

**Keywords:** cancer support, cancer survivorship, psychosocial support, rural health

## Abstract

**Purpose:**

Individuals diagnosed with cancer have extensive and often unmet psychosocial support needs. We established a partnership between the University of Minnesota and Gilda's Club to identify survivorship issues, unmet psychosocial support needs, and barriers to receiving cancer support in rural Minnesota.

**Methods:**

We conducted six focus groups and 16 interviews (41 total participants) between November 2022 and January 2024 among cancer survivors living in rural Minnesota. Structured interview guides included questions about survivors’ definition of cancer support, what community support is desired and available, and barriers to obtaining support. Transcripts were analyzed using inductive thematic analysis.

**Findings:**

The mean participant age was 57.1 ± 13.4 years; the majority were female (68%), non‐Hispanic White (95%), and college graduates (58%), and they represented many cancer diagnoses, with hematologic (20%) and breast cancers (17%) most frequently reported. Most (73%) were under surveillance (median 4 years from diagnosis). Many participants mentioned extensive travel burdens due to lack of local care, and virtually all participants agreed emotional support was critical. Over half (56%) of participants wished for peer support that they did not have, and 44% said their cancer information needs were insufficiently addressed. Some emphasized that having nurses facilitating care coordination and options for local care made care feel more personalized. Participants identified virtual options for cancer support as potentially beneficial, particularly when meeting in person was not possible.

**Conclusions:**

Lack of peer support, lack of local care, and travel burdens are significant concerns among rural cancer survivors. Participants expressed positive views about their rural residence and mentioned alternatives and rural strengths such as virtual support options, help from nurses, and caring relationships in their communities.

## INTRODUCTION

An estimated 20% of the U.S. population resides in rural areas,^1^ yet only 3% of medical oncologists practice in rural communities, and over 70% of U.S. counties lack a medical oncologist.^2^ In Minnesota, rurality is higher than the national average, with 27% of the population classified as rural.^3^ Rural patients face reduced access to essential medical care and support services for their cancer needs.^4^ Cancer incidence is higher in metropolitan populations, but rural counties experience higher mortality rates across all cancer types.^5^

Distressing psychosocial needs after a cancer diagnosis, including mental, emotional, social and spiritual needs of individuals with cancer and their families, are often unaddressed. Filling that gap, cancer psychosocial support programs empower patients by giving them access to services such as counseling, education, and support groups.^6^ Cancer support groups enable individuals and families to connect with others in similar circumstances, creating a community around shared experiences of dealing with cancer.^7^ The use of support services following a cancer diagnosis can lead to positive outcomes such as improving trust of and communication with providers, advocating for oneself, experiencing fewer physical and emotional health detriments, and improved overall quality of life.^8^, ^9^, ^10^

There is limited information regarding specific unmet support needs of individuals living in rural areas and what barriers rural cancer patients face in obtaining psychosocial support during their cancer journey. Gilda's Club Minnesota, a nonprofit organization that offers free psychosocial support for anyone impacted by cancer, including patients and their family members, is seeking to expand their services into rural Minnesota. In alignment with the Cancer Support Community Model, services offered by Gilda's Club Minnesota include weekly and monthly support groups, education, social support and gatherings, healthy lifestyle classes, information, and referrals for additional services.^11^ We established a partnership between researchers at the University of Minnesota and Gilda's Club staff members to conduct a research study to (1) identify survivorship issues and unmet psychosocial support needs among rural Minnesotan cancer survivors and (2) identify barriers to receiving cancer support in rural Minnesota and perceived opportunities to guide Gilda's Club future programming. Results of this study will not only guide Gilda's Club Minnesota but also provide data to identify efforts to support rural cancer survivors across the United States.

## METHODS

### Study participants and recruitment

We recruited individuals with a history of cancer living in rural Minnesota to participate in a qualitative study (focus groups and interviews) between November 2022 and January 2024. As a community‐led and community‐based project, the study and recruitment strategy were designed in collaboration with Gilda's Club leadership and with the Minnesota Cancer Clinical Trials Network (MNCCTN), a network of 24 clinics across five different health systems funded by the Minnesota legislature to improve access to cancer prevention and clinical trials in rural Minnesota. During initial recruitment strategy discussions, we learned that the name “Gilda's Club Twin Cities” ‐ the original name of Gilda's Club Minnesota ‐ could potentially deter individuals from participating in the study, as it might be perceived as exclusive to the Minneapolis/St. Paul metropolitan area, leading  rural Minnesotans to feel excluded. We shared this feedback with Gilda's Club leadership and with their support used a modified version of the logo for recruitment purposes, which minimized the emphasis on the name and location. Advertising flyers were placed in cancer clinics of the MNCCTN across the state. We also invited rural Minnesotans with a history of cancer within the MHealth Fairview system through a one‐time electronic medical record (MyChart) recruitment message. MHealth Fairview serves individuals primarily from Minnesota.

Inclusion criteria included age ≥18 years, residence in rural Minnesota, ability to read/write in English, and willingness to provide voluntary informed consent. To identify rural communities in Minnesota, we used Rural Urban Commuting Area (RUCA) codes, which classify areas in the United States based on how rural or urban they are taking into account population size and commuting patterns.^12^ We focused on zip codes in Minnesota representing codes 4–10 (large rural towns through remote rural areas); all participants self‐identified as living in rural areas. Interested individuals completed an online screener or called a study coordinator at Gilda's Club to confirm eligibility. The study coordinator followed up by phone to discuss study details and complete the remote electronic consent process. The University of Minnesota Institutional Review Board approved this study (STUDY00016378), and all participants provided remotely electronically signed written informed consent utilizing Research Electronic Data Capture (REDCap) eConsent.^13^, ^14^

### Study procedures

The initial plan was to conduct six to eight focus groups; however, due to difficulties in scheduling and participant preferences, we allowed individuals to choose between focus groups or one‐on‐one interviews, offering online and telephone options. Prior to the focus group or interview, participants completed a brief survey to collect demographic and clinical characteristics.

The University of Minnesota research team and Gilda's Club staff co‐developed the focus group and interview guides (Table 1). All staff were trained in conducting ethical human subjects research and implementing qualitative research.

**TABLE 1:** Focus group and interview questions.

How has your cancer impacted you in your life? This might include more than one issue related to your cancer. Please think about cancer support that you have received, and cancer support you would like to have. How do you define cancer support? What kind of cancer support is available in your community? What kind of emotional, social, or other cancer support would you have liked to have but did not receive? What are barriers to getting cancer support to address your unmet needs? What is your view on emotional cancer support? Is it important to you? Is there any stigma associated with it? How available is emotional cancer support? What kind of cancer support offers would you be willing to use on remote options (Zoom, etc.), which ones only in person? Do you think the fact that you do not live in a big city has shaped your cancer experience, including cancer support? If so, how? Do you have a cancer community in which you feel “you fit in?” How could psychosocial support from an organization such as Gilda's help you to feel less alone in your cancer journey? What are other cancer support services that you would like to use that we have not talked about?

Focus groups were approximately 90 min in length; individual interviews were approximately 30‐ to 45‐min long. Sessions were conducted on Zoom or by telephone. S.J. and R.A. were the session moderators/co‐moderators for the focus groups, and S.J., R.A., and M.G. conducted interviews. They followed the question guides, asked clarifying and follow‐up questions as appropriate, and summarized the conversations for participants at the end of each session. Participants confirmed their agreement to participate and to be recorded prior to the start of each session. Sessions were digitally audio‐ and video‐recorded. All participants received a $100 gift card following their session.

### Data analysis

Recordings captured via Zoom were initially transcribed using its AI Companion, manually reviewed, corrected and anonymized, and presented verbatim; phone interviews were manually transcribed verbatim and anonymized by the research team. Two University of Minnesota team members analyzed transcripts using an inductive thematic analysis approach (P.J., X.L.).^15^ They each listened to and watched the audio and video recordings, read the transcripts, and identified initial codes. They then reviewed the codes together, and then each separately reviewed the transcripts again, applying the codes to the transcripts. The codes and associated excerpts were reviewed and then grouped into themes, which the two analysts also discussed. The research team and Gilda's Club staff reviewed the themes and agreed upon the final categorization of themes and subtopics. We continued conducting focus groups and interviews until we reached thematic saturation; we did not glean additional themes or subtopics from the last focus group or the final three interviews. The themes are structured in alignment with the Cancer Support Community model.^11^ Representative quotes from participants relevant for each theme are provided.

## RESULTS

**FIGURE 1:**
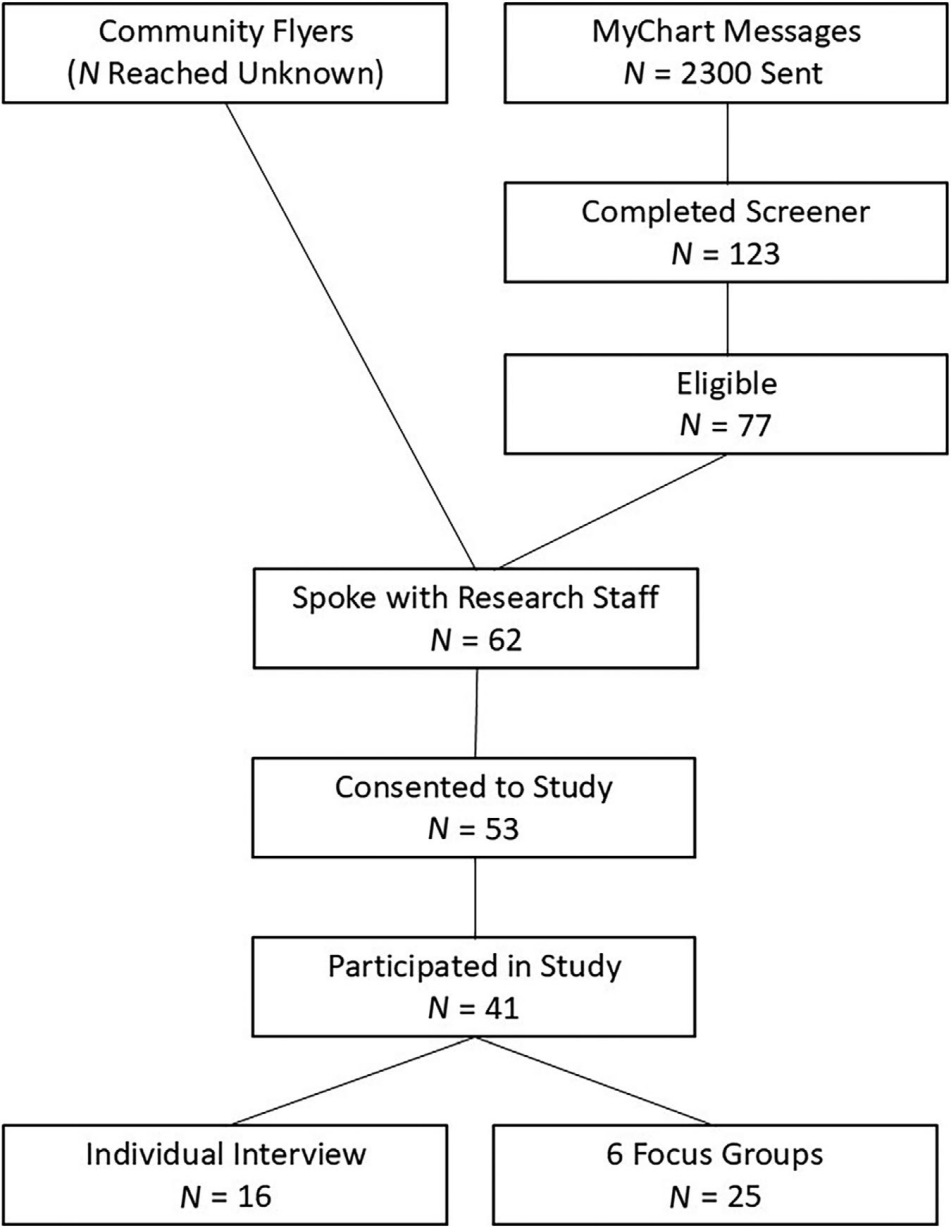
Participant flow chart.

Using a combination of clinic and community study flyers and sending 2300 MyChart messages to potentially eligible individuals, a total of 123 individuals completed a screener to determine eligibility, 62 spoke with study staff, 53 completed the consent process, and 41 ultimately participated in either a focus group (six groups, 25 individuals total) or interview (16 individuals; Figure 1). The mean age of participants was 57.1 ± 13.4 years (Table 2). Most participants were women (*N* = 28; 68%), were non‐Hispanic White (*N* = 39; 95%), had a college degree (*N* = 23; 58%), and were married or partnered (*N* = 27; 66%). The majority (*N* = 30; 73%) of participants reported having grown up in rural communities, and most (*N* = 25; 61%) stated that rurality was a part of their identity. The most frequent cancer diagnoses were hematologic (*N* = 8; 20%) and breast (*N* = 7; 17%) cancers, with 5 (12.2%) participants reporting more than one cancer type. T ashe most common among women were breast and hematologic cancers, and the most common among men were prostate cancer and melanoma. Approximately half (*N* = 22; 54%) were diagnosed with stage I or II disease, and most (*N* = 30; 73%) were currently in surveillance and not receiving active treatment.

**TABLE 2:** Participant demographics, cancer clinical characteristics, and self‐reported rural residence and identity (*N* = 41).

Characteristic	*N*	Mean/median
Age (years), mean ± SD	41	57.1 ± 13.4
Time since diagnosis (years), median (range)	40	4 (0–22)
	*N*	%
Gender		
Female	28	68.3
Male	13	31.7
Race/ethnicity		
Hispanic	1	2.4
More than one race (Black, White)	1	2.4
White	39	95.1
Education		
High school graduate	3	7.5
Some college/AA degree/vocational school	14	35.0
College graduate	23	57.5
Missing	1	
Marital status		
Never married	5	12.2
Married/partnered	27	65.9
Widowed	3	7.3
Divorced/separated	6	14.6
Annual household income		
<$50,000	9	22.5
$50,000–$99,999	12	30.0
$100,000+	15	37.5
Prefer not to answer	4	10.0
Missing	1	
Employment status		
Not working	9	22.0
Working full‐time	15	36.6
Working part‐time	5	12.2
Retired	12	29.3
Grew up in small town or rural area		
No	11	26.8
Yes	30	73.2
How much is being from a small town or rural area part of your identity?		
A great deal	13	31.7
Quite a bit	12	29.3
A moderate amount	7	17.1
A little	5	12.2
Not at all	4	9.8
How well does the term “rural” describe you?		
Extremely well	4	9.8
Very well	11	26.8
Moderately well	16	39.0
Slightly well	4	9.8
Not well at all	6	14.6
Cancer site		
Brain	2	4.9
Breast	7	17.1
Gastrointestinal	3	7.3
Gynecologic	4	9.8
Head and neck	3	7.3
Leukemia/lymphoma/other blood cancers	8	19.5
Prostate cancer	3	7.3
Multiple cancers	5	12.2
Melanoma	3	7.3
Neuroendocrine	1	2.4
Sarcoma	1	2.4
Thyroid	1	2.4
Cancer stage		
Early stage	22	53.7
Advanced stage	11	26.8

**TABLE 3:** Social support, with representative quotes by subtheme. Participants’ ages are shown as age groups to protect anonymity.

Theme	Quote
**1. Social support**	
Peer support	“The person I would've appreciated talking to most would have been someone who'd gone through the same or similar treatments, someone who could really understand the fears and anxiety.” (Female, 60–69) “I would be fine virtually or even if I could just text a person who has gone through this and just say, “Hey, I just finished my immunotherapy, and this is how I'm feeling,” or like “Christmas is coming up, and that's the anniversary of me starting chemo and these are the emotions that are coming,” and just like, “I found out I can't donate blood anymore.” Just like small things that actually feel like are big things, so to quickly answer your question, in‐person would be awesome, but I would be fine having a text, just like a friend, you know? A cancer‐mentor‐friend.” (Female, 30–39) “When I got back home [from staying in metropolitan area for treatment] there, we, and in [name of rural town], we have a local support cancer support group. But it was very much, much more people that weren't my age. […] But what I would have really liked is somebody that understood this particular cancer, that, the fact that it's considered incurable, there is no walking away from it.” (Female, 50–59)
Loneliness and isolation	“I kinda feel like I'm on my own with it.” (Female, 40–49) “I don't feel like a lot of people who haven't gone through cancer get it… So, when I finished treatment, everyone was really happy about it and I just felt very flat. And I didn't know why I felt so flat, and then I talked to ladies in my support group, and they said, “Oh, we felt the same way too.” And so, it just, feels kind of like I've been changed and no one really understands the different person that I am, and I don't get it either, but I just feel like I am different, and people don't… I don't feel like I click with people the same way that I did before, and so that was a completely unexpected and really difficult change.” (Female, 30–39)
Support groups	‘On the emotional side of it, being able to talk things through sometimes is really helpful. I didn't experience big issues for myself, but I mean, sometimes just being able to come together as a group and, it does not need to be a woe‐is‐me kind of thing, but you know, just share it. “What are you going through? What are your thoughts? What are your fears?”’ (Male, 60–69) “On like some [online] sites there, it tends to be a lot of venting, which is hard for me to hear other people's pain like I, it's hard for me to hear other stories, like I'm very empathetic. So that's difficult.” (Female, 50–59) “We live two and a half hours north of the cities, so it's just not practical for me to go down to the cities, so I'm too very thankful for the zoom option.” (Female, 50–59) “I'm more on person to person, person, you know. I like to see the person who I'm talking to. Zoom is good, too. It's good, too, but I think if you do zoom, you're leaving the personal contact out of it. I mean, I like to be on one on one.” (Male, 60–69)

**TABLE 4:** Emotional, psychological, and spiritual support, with representative quotes by subtheme. Participants’ ages are shown as age groups to protect anonymity.

Theme	Quote
**2. Emotional, psychological, and spiritual support**	
Emotional support	“The after is kinda scary, too, because it's never, you never passed it really, like it's never really over. It's just kinda like, how do I live with it now? And so maybe some more just emotional support even that way would be helpful. Because you're not really done when you're done with treatment.” (Female, 40–49) “I think you have to have your family by you, and if you don't have a family by you, then you need to seek out that support in some way. Either it's a group or through your, you know if you go to church, through your church group, stuff like that. I think if you're dealing with cancer, you need a, you need that support. And it's, and I think it builds your, it makes you stronger when you feel you have that support behind you.” (Male, 60–69) “My wife is just fantastic, and I don't know that I could say I would need some kind of support network.” (Male, 50–59) “I need some emotional help, and I feel very alone in my community. I feel alone in my family. I was estranged from my mom and sister like before I was diagnosed. And so it's, so I didn't have a lot of family support that way. I was divorced. I wasn't dating.” (Female, 50–59)
Stigma of seeking support	“Being a rural Minnesotan, you know, especially if you're a certain stock you don't necessarily like, just open up easily to, to just everybody.” (Female, 70–79) “I suppose there's just a natural stigma of needing help that you're not, you know, strong enough to go through it on your own, but, but at the same time, I, I mean, nobody knows what you're going through until you go through it yourself. So I don't really, I don't really care what people think at this point.” (Female, 40–49) “There was a support group there, and, but we never seeked it out, you know. That's the way families are, you know, some families do, and some families don't. We're the families that didn't.” (Male, 60–69)
Lack of privacy in small community	“My issue is […] that you know everybody. And some people like that. I do not.” (Female, 50–59) “If I was in a very large community and went to a support group, I wouldn't know anybody, so I probably could speak more freely if I felt like it.” (Female, 60–69) ‘I did check with my local hospital [about support groups] and they said, the social worker said, “oh yes, we have a support group, please come join.” Well, I was the only female in a group of… I think it was 3 men. And I found it, for me personally because of the type of cancer, uterine cancer, I was so uncomfortable in that. So, I participated one night and said, “thank you very much, but I think I'd rather find a group regarding gynecological cancers.”’ (Female, 60–69)

**TABLE 5:** Informational support, practical and instrumental support, resources for symptom management, and unique strengths of rural communities, with representative quotes by theme. Participants’ ages are shown as age groups to protect anonymity.

Theme	Quote
**3. Informational support**	
Information needs	“Just speak to me like I have no clue, cause I don't. So that's probably, initially the biggest part of cancer care to me was just answers, information.” (Male, 60–69) “And I feel my urologist is really great […] when he, when he explains stuff to me, I don't have to say to “Please come on down from your ivory tower, I'm not a doctor, I'm a lay person.” He's really good about, you know, being very, he can explain things in a layman's term. So that's important to me as a person having cancer.” (Male, 80–89) “[…] our health care system is kind of a mess. And people really need to understand and have support for advocating for themselves. You know, sometimes you kind of have to just raise your voice and say, wait a minute, you know more, here's, what here's what I need.” (Male, 70–79)
Potential solutions	“When I had the initial diagnosis, the woman that helped walk through, you know, went to all the meetings with me. Man, without her, I don't know what I would have done. It wasn't so much emotional help. But I was only hearing like “Hey, with your cancer, breast cancer!” You know, I'd hear those big awful words and get really scared, and she would sit and write everything down at the end of the meeting and ask me if I had more questions. And I didn't, but then when I get home and calm down a little bit from… from the shock and awe of everything, she was always there and that was great. And I think that was just a service that the hospital or the organization provided, which was, like, super. I don't know what it would have done without it, to be honest.” (Female, 60–69)
**4. Practical and instrumental support**	
Travel, financial, and time burdens	“I would say in the rural area, if you can't drive, there's a barrier. If you're poor, there's a barrier. If you don't have people to help you, there's a barrier.” (Male, 60–69) (Participant who is also a caregiver of his father who also has cancer): “We drive down to the cities every couple of weeks for his treatments, and like I didn't know there was a place to stay, and like cause we just, it's about 14–15 hour round trip with after his treatment. And so I drive down, drop him off, he goes in because a lot of it was during the Covid, so I couldn't go in, and, yeah, wait for him to be done and then come back home. And yeah, we'd leave at 7 and get home between 7 and 10.” (Male, 40–49)
Practical support	“The American Cancer Society Hope Lodge. I don't know what I would have done without it, because it was free lodging for the next 8 weeks I was there. The bad side of the Hope Lodge was that meant I definitely had cancer because they don't let you in if you don't have it. That was huge support. The other part of the part of that support is being with other cancer survivors and their caregivers. I, I can't even I, I, I just there's still no words, just, it was such a positive experience to be there with all those families and people that were going through various types of cancer, various stages, all ages, all ages of people. And so that was just the most supportive environment I could have been. And I, I think without it I would not have accepted what was happening.” (Female, 50–59)
**5. Physical and symptom management**	
Few available local resources	“I don't think there's a whole lot of anything [cancer support]. I've been looking all over and there's nothing.” (Female, 20–29)
**6. Unique strengths of rural communities**	
Strengths of rural communities	“There's just something to be said about getting your care from the same people in your community […] I don't know. I think when it comes to just that small town living or rural life, especially when it's people who aren't just coming and going, you know, they're here for decades. There's, there's just a level of trust and connection that I do really appreciate about that.” (Female, 40–49) “Going to the cities for me […] it'd be very hard for me to move after living in a rural area all my life, and that would have been extra, probably would have been worse than the cancer for me. I hate to say that I don't, you know, the nearest stop light from my house is 12 miles away, so definitely, definitely a different lifestyle for me, you know. Not my cup of tea.” (Male, 70–79) “In order to be closer to my family, my daughter, and my son‐in‐law, whatever the disadvantage medically [easier access to care option] does not outweigh the advantages that I have for living here, as far as being close to family.” (Male, 80–89)

### Social support (Table 3)

#### Peer support

Most participants (*N* = 23; 56%) stated they would like to have, but did not have, peer support. Peers were broadly defined as individuals with shared lived experiences that could relate to the situation of the patient. Participants had differing criteria for defining peers. For example, some participants defined peers along specific shared cancer characteristics, such as having the same type of cancer, being able to relate to living with “incurable” cancer, having gone through similar treatments, or having experienced similar symptoms or side effects. Other participants emphasized the importance of demographic similarities such as gender, age, and life stage; for example, almost all younger participants in the study said they would like to connect with cancer patients in their age group and at similar life stages because having cancer at young age is rare, with unique struggles, and therefore a lonely experience. In contrast, while also using an aged‐based peer definition, an older participant who lived in a retirement home said that connections to other residents with ailments, not necessarily cancer, created an environment where people could relate to one another based on the general shared experience of struggling with health issues related to age. A few participants specifically pointed out that they perceived HIPAA restrictions and lack of efforts of clinics to connect cancer patients with each other as barriers because it was highly unlikely they would meet cancer peers in their own small and remote communities.

#### Loneliness and isolation

When asked, the majority of participants said they had no cancer community. Of note, the way participants answered this question differed from the other questions. For more questions, participants provided nuanced explanations that specified and modified answers to ensure clarity In contrast, when asked whether participants felt they had a cancer community, they responded simply and unambiguously “no” without further modifying comments. Most participants interviewed reported they had no cancer support services available or were unaware of the cancer support services available, if any were, in their small remote communities. Some participants shared that people they had been close to before their cancer diagnosis could no longer relate to what they had been through or how cancer had changed them. This disconnection led to a sense of loss and mourning of formerly close relationships. However, there was also a group of participants who reported they were coping well, either because they had all the supportthey needed or felt they did not need any support, and therefore did not feel a need to engage with a cancer community.

#### Support groups

Participants' experiences ranged from attending in‐person groups to engaging in online or virtual formats. Others shared opinions based on assumptions about how support groups might work or affect them emotionally. While some acknowledged the benefits, several participants noted they were not emotionally prepared to face others’ suffering, particularly while managing their own challenges. Some expressed interest in more “lighthearted” support options—focused on uplifting survivorship stories or shared social activities like exercise, crafts, or other group experiences that do not center exclusively on discussion of cancer.

With regard to the modality of cancer support groups, most agreed that cancer support would ideally be in person as virtual options do not create the same connection between people. Other reservations about virtual cancer support options included technological difficulties and lack of trust in the confidentiality of the technology. Others simply said they were tired of virtual meetings. At the same time, most participants agreed that virtual meetings were a welcome second‐best alternative if in‐person support groups were not realistic because people lived in remote places, were too sick to travel, or were immunocompromised. A few participants explicitly advocated for a mix of virtual and in‐person options for cancer support groups in rural areas. Others pointed out that virtual meetings allow for greater anonymity, in contrast to in‐person meetings, which would help them feel more comfortable discussing their personal experiences and needs.

### Emotional, psychological, and spiritual support (Table 4)

#### Emotional support

Participants overwhelmingly agreed that emotional support is important when one has cancer, and some emphasized that family members without cancer also needed support. Participants differed in whether they felt that their emotional support needs were being met. Many said that they received sufficient support from family and friends, while others noted that, although they had some support from family and friends, they still needed additional emotional support. A few reported having little or no  emotional support from any sources. Some participants (*N* = 9; 22%) said they were coping well and emphasized that they did not need emotional support, either at all or beyond what was already available to them, typically from a partner or family members.

#### Stigma of seeking support

Participants expressed a range of opinions on whether stigma is associated with seeking cancer support. Some stated that while stigma existed, it was irrelevant and easy to ignore, and they would still seek out the support they needed. Others felt stigma was more pronounced in rural communities, where ‐ as one participant described ‐ people are of a certain “stock” and may be less inclined to open up to others or ask for help, even when support could be beneficial. Others believed that although stigma existed, it was gradually diminishing as seeking support was becoming more normalized, including in rural areas. A few participants said they did not believe there was a stigma associated with seeking cancer support.

#### Lack of privacy in small community

Some participants’ hesitancy to discuss their cancer with others was specifically contextualized within a perceived lack of privacy in small rural communities where “everybody knows everybody,” creating barriers to seeking support. Some participants reported having reservations about sharing themselves with others and being vulnerable and said that they would prefer a more anonymous setting. A few participants mentioned hesitancy talking about one's bod, for example, about one's breasts. One participant said that since her cancer affected an intimate, in her case, gynecologic, body part, she would not join a mixed‐gender support group.

### Informational support (Table 5)

#### Information needs

Many participants emphasized they had previous or continuing information needs that were insufficiently addressed. Participants wished for more proactive communication and explanations from medical teams about what to expect; they wanted transparency, honesty, and comprehensive information. They added the importance of explaining what patients might expect during and after treatment and addressing some patients’ erroneous beliefs that cancer is automatically a death sentence. Several patients emphasized that in addition to what information medical teams provide, how that communication occurred was important. Participants described that some people “know how to listen,” while others do not. Several participants emphasized that information was not always provided in ways that were understandable to them, especially at the time of initial diagnosis when patients had to process a lot of information and make difficult decisions. Furthermore, care coordination and information flow between providers and health care systems were not always smooth.

To address information needs that were inadequately addressed in their cancer care, some participants recommended connecting cancer patients with individuals who could help them navigate and fully understand the information. Suggestions included nurse navigators and nurse “translators,” especially at their initial diagnosis. Peer‐to‐peer support, where cancer patients are connected to cancer peers who had similiar experiences, was suggested as a way to help new patients navigate their cancer journey. To address gaps in rural health care infrastructure, some suggested traveling nurses, for example, to facilitate blood draws and lab work prior to chemotherapy appointments from one's home and hence reducing some of the rural care travel burdens.

Some participants described receiving support from nurses who helped translate complex medical terminology into language that patients could understand, facilitated communication by asking and answering questions, and were available for questions that came up outside of clinical settings. Others stated that many cancer patients may not know what they need or what support services are available to them. They suggested that an organization like Gilda's Club could play a role in helping cancer patients learn to advocate for themselves. The importance of self‐advocacy was emphasized by several patients, who felt that cancer patients had to be outspoken if they wanted to have their needs addressed. They stated that health care systems were not designed to automatically provide cancer patients with information about support options. Participants also noted that some people do not know what support they would benefit from and are reluctant to ask. Those who said they spoke up for themselves acknowledged that not everybody does or can do the same.

### Practical and instrumental support (Table 5)

#### Travel, financial, and time burdens

Almost all participants described travel as a significant burden due to long distances between their home and cancer care facilities. However, this varied based on participants' locations, mobility, and the availability of nearby care options. Those living in rural areas closer to population centers with medical facilities found travel burdensome but manageable. In contrast, others reported living several hours from care, requiring them to either plan for long days or arrange overnight stays near the clinic. Travel for cancer care also introduced additional financial and time‐related stressors, including the cost of gas and lodging, childcare responsibilities, and excess mileage on vehicles.

#### Practical support

Multiple participants mentioned the impact of small, concrete everyday support they had or would like to have, for example, help with household chores and physical tasks, driving to appointments, meals, and sending cards. Some participants suggested services to address the remoteness of rural spaces, such as transportation services, financial services, and options for free accommodation at cancer clinics to avoid some expenditures and travel burdens. Several participants mentioned the American Cancer Society Hope Lodge near the University of Minnesota cancer clinic where cancer patients and their families can live for free during treatment. While all of those who used the Hope Lodge stated it reduced cancer‐related expenses for them, one participant additionally mentioned that staying at the Hope Lodge had also meant she got to connect with other people with cancer, which automatically provided some peer support, and that Hope Lodge offered some non‐oncology holistic services that were helpful.

### Physical and symptom management support (Table 5)

While we did not explicitly ask about support for symptom management or physical concerns, the lack of availability of local cancer care options in small communities was emphasized by most participants. Participants listed non‐oncology complementary and integrative care services that were typically unavailable to them in their communities, such as speech therapists, nutritionists, trainers, chiropractors, massage therapists, occupational therapists, acupuncture, exercise programs, and palliative care. Answers were nuanced if participants lived in medium‐sized towns where some more care options were available. Many participants said they simply did not know what was locally available.

### Unique strengths of rural communities (Table 5)

Participants emphasized strengths of rural communities. Some participants said that if one can get health care in rural communities, it is often more personal than in larger places because rural communities are often characterized by being less anonymous and having more connections and networks within one's community. With some exceptions, participants said they trusted their local physicians and medical teams. Some participants also said they could not imagine living anywhere else because they were attached to the places where they lived, and these sentiments outweighed challenges regarding access to care and support. Several participants also mentioned they had had critical support from their local faith communities.

## DISCUSSION

The objectives of this qualitative study were to identify survivorship issues and unmet psychosocial support needs among rural Minnesotan cancer survivors and identify barriers to receiving cancer support in rural Minnesota and perceived opportunities to guide Gilda's Club future programming efforts.

Many themes we identified regarding cancer care and support needs in rural communities have been previously identified. Individuals living in rural communities face worse survival rates following a cancer diagnosis compared to those in urban areas.^16^ Our research confirmed findings from other studies that reported financial barriers, poor or lack of insurance coverage, transportation issues, and lack of available local care^17^ as contributing factors accentuating the disparity in cancer survival rates between those in rural and urban settings. Participants in our study emphasized that long travel for cancer care, and in some cases, having to stay near cancer care facilities overnight, increased financial and logistical burdens for cancer patients and their caregivers who often managed transportation.

Some of our findings have been less frequently described in prior studies and warrant additional emphasis. Most of our participants mentioned the positive impact of peer support from other individuals with cancer but that it was difficult or impossible to find such peers in their rural communities. Some participants also experienced disruptions or loss of close relationships with people who did not have cancer, noting that their cancer diagnosis led them to feel like their friends or family no longer understood them and their needs. Married or partnered males frequently referenced their wives/partners as their main source of support; however, females were less likely to report this and suggested support from their partner was not sufficient. Some participants called out failures of health care systems to help cancer patients find peers by not facilitating cross‐connections between patients, with some going so far as to call HIPAA regulations harmful because they prevented clinics from connecting patients with each other. While calling out these structural issues that make rural cancer journeys harder and that led participants to conclude that unless they self‐advocated, they would not get any cancer support, participants also suggested solutions. These suggestions included traveling nurses, medical transportation services, nurses as translators of medical jargon into information that patients can understand, and free accommodations near cancer treatment centers for those who need to travel far for cancer care such as the American Cancer Society Hope Lodge in Minneapolis. Support organizations such as Gilda's Club may fill other gaps, for example, by connecting cancer patients with peers and by enabling them to advocate for themselves.

Previous work has described that lack of privacy in small communities can be problematic in rural areas.^18^, ^19^ In our study, we additionally heard suggestions that some rural residents may find it harder to share cancer‐related concerns with others, while others felt they had strong social cohesion and faith communities. By connecting people with cancer who do not live in the same community, organizations such as Gilda's Club could help overcome some privacy concerns of cancer patients living in rural areas. Support organizations can also help caregivers, another vulnerable group mentioned by several of our study participants. Kent et al. identified that caregivers of rural patients with cancer report unique social needs in physical, financial, interpersonal, and service domains.^20^

Finally, in the conduct of this study we learned that the original branding of “Gilda's Club Twin Cities” was a potential barrier to study participation. Gilda's Club Twin Cities, upon review with their leadership team and consultants, officially changed their name to “Gilda's Club Minnesota” in January 2024 to reflect their commitment to serving the entire state. We feel this is an important finding and a reminder for support organizations to consider how they brand themselves, as it may affect their ability to reach their target audience.

The qualitative method approach to this study allowed for a detailed assessment of the psychosocial support needs of rural Minnesota cancer survivors and captured personal experiences. It is both a strength and weakness that we included survivors with different cancers and cancer stages and included individuals both near and further out from diagnosis and treatment. An additional limitation was the lack of diversity in our population; while rural Minnesota is primarily non‐Hispanic White, there are specific immigrant populations and native populations whose experiences may not be captured in our data. This study highlights the importance of and need for community‐engaged work.

In conclusion, rural cancer patients desire peer support that can be difficult to find in small rural communities. They experience exaggerated loneliness and time and financial burdens related to a cancer diagnosis and subsequent care, with fewer support options to address multiple cancer‐related needs and stressors due to rural infrastructure gaps. Yet, participants also noted strengths in their rural communities that should be supported and built on. To meet the needs of rural cancer survivors, solutions need to incorporate a partnership with community partners and both in‐person and virtual cancer support options.

## CONFLICT OF INTEREST STATEMENT

The authors declare no conflicts of interest.
